# Graphic cigarette pack warnings do not produce more negative implicit evaluations of smoking compared to text-only warnings

**DOI:** 10.1371/journal.pone.0194627

**Published:** 2018-03-15

**Authors:** Pieter Van Dessel, Colin Tucker Smith, Jan De Houwer

**Affiliations:** 1 Department of Experimental-Clinical and Health Psychology, Ghent University, Ghent, Belgium; 2 Department of Psychology, University of Florida, Gainesville, Florida, United States of America; Legacy, Schroeder Institute for Tobacco Research and Policy Studies, UNITED STATES

## Abstract

Graphic warnings (GWs) on cigarette packs are widely used internationally with the aim of reducing smoking behavior. In the current study, we investigated whether GWs influence implicit evaluations of smoking, a potential moderator of smoking behavior, as measured with an Implicit Association Test (IAT). Results showed that viewing a GW did not produce more negative implicit evaluations of smoking for daily smokers, occasional smokers, or non-smokers, compared to viewing a text-only warning. If anything, effects were in the direction of evaluations of smoking being *more positive* after certain participants (i.e., daily and occasional smokers) viewed a GW. We also did not find any beneficial effects of GWs on explicit evaluations of smoking. These results contrast with the observation that non-smokers and occasional smokers (but not daily smokers) believed that GWs would be more effective than the text-only warnings. We discuss implications and limitations of these findings and provide recommendations for improving the effectiveness of cigarette pack warnings on implicit evaluations.

## Introduction

Smoking is the leading cause of preventable death and disease worldwide [1]. Hence, understanding how we can efficiently prevent initiation and encourage cessation of smoking is an important research endeavor. One of the primary strategies of tobacco control efforts has been the placing of warning labels on tobacco products. These warning labels consist of text messages that are designed to inform people about the negative consequences of smoking such as ‘Cigarettes cause fatal lung disease’. Recently, many countries have started to require putting *graphic* health warnings on cigarette packaging. Graphic warnings (GWs) typically consist of a text message supplemented with a confronting color photograph, such as an image of blackened lungs or gangrenous feet, that is designed to invoke fear. Because fear can be an important motivator for behavioral change [2], GWs are often considered to be an effective tobacco-control strategy.

In line with this view, a recent meta-analysis, that included the results from 37 experimental studies, has shown that GWs produce better effects than text-only warnings on a number of important outcomes [3]. For instance, GWs attract and hold attention better, motivate people to think more carefully about the risks of smoking, and more strongly increase smokers’ intentions to quit smoking. Other studies examining the impact of cigarette pack warnings have further shown that GWs are typically perceived as more effective than text-only warnings and more strongly promote smoking cessation as well as prevent smoking initiation [4–8]. Another recent review assessed the long-term outcome of the national implementation of GWs in 20 countries [9]. This review showed beneficial effects of GWs on people’s knowledge about smoking harms, knowledge of smoking cessation telephone lines and calls to these lines, as well as reductions in actual smoking behavior.

In recent years, however, a number of researchers have expressed concern about the effectiveness of graphic warnings [10–12]. One important critique is that GWs might (strongly) influence smokers’ intentions to quit smoking but these effects do not always translate into behavior. This accords with the observation that correlations between self-reported intention and behavior are not consistently strong with habitual behaviors such as smoking [13–14]. Hence, the modification of actual smoking behavior might sometimes depend on changes in other determinants of smoking. By investigating the extent to which GWs may influence certain moderators of smoking behavior we might gain important information that can help improve GWs or other cigarette pack warnings.

One important moderator of smoking behavior is a person’s attitude towards smoking. Cigarette pack warnings are issued in order to provide information that convinces people that smoking is bad. The desired outcome of a person reading these messages is that he or she will evaluate smoking more negatively and that this will cause smokers to reduce their smoking behavior and non-smokers to refrain from picking up smoking. Importantly, implicit evaluations of smoking (i.e., the spontaneous evaluative responses to smoking-related stimuli) might have a connection with smoking behavior in this respect. Previous research has provided evidence that implicit evaluations of smoking can be a good predictor of smoking initiation [15], quitting behavior [16], and abstinence or relapse after quitting smoking [17–18]. Implicit evaluations of smoking are sometimes found to be a reliable correlate of these various aspects of addictive behavior even when controlling for changes in more controlled, explicit evaluations of smoking (though the observed relation may depend on several moderators) (see [19], for a review). For these reasons, it is often argued that it is important to target implicit evaluations of smoking in order to reduce smoking behavior [14,20].

Cognitive theories of evaluation have traditionally assumed that (a) implicit evaluations reflect the automatic activation of associations between representations in memory (for a review, see [21]) and (b) changing these associations requires many experiences (e.g., [22–23]). As a result, interventions to change implicit evaluations of smoking have mainly used paradigms in which stimuli are repeatedly paired with valenced events (e.g., with the presentation of valenced stimuli: [24] or with the performance of approach-avoidance actions: [25]). Importantly, however, recent studies have provided substantial evidence that, under certain circumstances, implicit evaluations can actually change very rapidly on the basis of novel information [26–28]. For instance, studies in the context of smoking have found that smokers sometimes exhibit more negative implicit evaluations of smoking after reading anti-smoking messages [29] or after viewing a short anti-smoking Public Service Announcement [30]. These results accord with recent evaluation theories which assume that the acquisition of propositional information can have an immediate impact on implicit evaluation [31–34].

There are thus theoretical and empirical reasons to believe that even a single exposure to GWs can, at least in principle, influence implicit evaluations of smoking. In the current study, we examined empirically whether GWs actually produce these effects. To this end, we asked participants to first read one anti-smoking message which either did or did not include a graphic image and then complete an Implicit Association Test (IAT; [35]) measuring implicit evaluations of smoking. We decided to use the IAT because this is currently the most widely used method to measure implicit evaluations and because (changes in) IAT scores are sometimes found to be a good predictor of (changes in) smoking behavior [15]. Participants also completed an explicit measure of their evaluations of smoking (i.e., liking ratings) and indicated how effective they considered the anti-smoking warning they had seen. Note that, even though the focus of our study is on implicit evaluations, we included explicit evaluations and effectiveness ratings in the interest of comparison. In line with previous studies [3], we expected that participants would (a) rate GWs as more effective than text-only warnings and (b) exhibit more negative explicit evaluations of smoking after viewing GWs. Given the previous observations of both beneficial and unwanted effects of anti-smoking messages on implicit evaluations [28], we did not have strong predictions regarding the (direction of) effects of GWs on implicit evaluations of smoking. We investigated effects of GWs for non-smokers, occasional smokers, and daily smokers separately because previous studies have shown that these groups can respond differently to anti-smoking messages [36]. We recruited a large number of participants in order to maximize statistical power to find effects of this single exposure to the warning in each of the groups. We stopped the data-collections when at least 1000 smokers had completed all measures of the experiment. As a result, we had sufficient statistical power to detect small effects even for the subset of participants that were daily smokers (power > .80 to detect an effect size of *d* = 0.22). All data were collected in one shot without intermittent data analysis.

## Materials and methods

### Participants

A total of 7757 English-speaking volunteers participated online via the Project Implicit research website (https://implicit.harvard.edu). Participants were citizens of 103 different countries (77.54% US, 5.81% Canada, 3.19% UK, 1.47% Australia, all others <1.00%). In line with the standard treatment of Project Implicit data [29], we excluded data from participants who (a) did not fully complete all questions and tasks (1422 participants; i.e., 18.33%), (b) had IAT error rates above 30% across the entire task (110 participants; i.e., 1.74%), (c) had IAT error rates above 40% for any one of the four critical IAT blocks (380 participants; i.e., 6.23%), or (d) were faster than 400 ms on more than 10% of the IAT trials (12 participants; i.e., 0.21%). Analyses were performed on the data of the remaining 5833 participants (64% women, mean age = 29, *SD* = 13). Note that including the data from all participants in the analyses did not result in any shift in significance for any of the reported effects. A full description of these results can be found at https://osf.io/g2tr7. At this online repository we also provide a link to the online study as well as all data of the study and data analysis scripts.

In line with previous research [36], we divided the sample of participants on the basis of smoking behavior by those who reported that they never smoke (4119 [70.61%] non-smokers), those who reported that they smoke less than one cigarette per day (1167 [20.01%] occasional smokers), and those who reported that they smoke at least one cigarette per day (547 [9.38%] daily smokers).

Ethical review and written approval was granted by Ghent University’s Ethics Committee. Prior to accessing the online study, respondents were directed to an Information Sheet setting out the study purpose, intended data use, and their rights as research participants. They were given a researcher’s contact details, should they have any questions, and advised that completion of the survey would be assumed to imply consent.

### Stimuli

We used 49 different cigarette warning messages as stimuli. First, we included the 36 graphic warning messages proposed by the US Food and Drug Administration (FDA) for inclusion on United States (US) cigarette packs. These messages consist of one of nine possible text messages and a color photograph matched to the text. All 36 messages were under consideration for inclusion on cigarette packs at the time of the study. Second, we included the four text-only warning messages that were currently in use in the US at the time of the study as well as the nine text messages proposed by the FDA without the color photographs. Participants were randomly assigned to one of the 49 messages (36 GWs and 13 text-only warnings).We opted for this large variability in GWs and text-only messages to allow for generalizability (i.e., possible effects are less likely to be due to one message that stands out as being very effective or un-effective) and to be able to test which messages within text-only and GWs might be most effective. All analyses were also performed when including only 9 of the 36 GWs (i.e., the GWs eventually selected by the FDA). Results of these analyses did not change the significance level of any of the reported effects.

The proportion of participants categorized as non-smokers, occasional smokers, and daily smokers was not significantly different for participants who read a GW or a text-only warning (graphic: 70.59%, 19.42%, 9.99%; text-only: 70.62%, 20.22%, 9.15%), *χ*^2^s < 1.83, *p*s > .17. We also did not observe any significant differences between participants who read a graphic or text-only warning in terms of sex, age, education level, race, or country of residence (US/non-US).

### Procedure

All participants were first informed that the FDA is currently considering new standards for warning labels on cigarette packaging and that they have put out a list of potential statements and images, asking for public comments on these labels. This information was true at the time of the study. Participants were told that they would see one of the potential labels and were asked to pay close attention to what they would see because they would be asked questions about it throughout the rest of the study. Then participants were shown one of the 49 possible cigarette warning messages (text warnings presented in black font; GWs presented in full color) on a screen with white background and with the following instructions presented directly below the message:

Please look carefully at the potential warning label above. Imagine that you see this image on a cigarette package. When you have finished thinking about it, press 'Continue' to go on with the study.

Half the participants completed an IAT measuring implicit evaluations of smoking immediately after reading the warning label. The other participants first reported their explicit liking of smoking. The IAT procedure followed that of previous studies [29]. Participants categorized 14 attribute words (e.g., happy, evil) as ‘positive’ or ‘negative’ and 12 images of smoking-related (e.g., cigarette, man smoking) or non-smoking related stimuli (e.g., pencil, man blowing a whistle) as ‘smoking’ or ‘not smoking’. The IAT consisted of three practice blocks and four experimental blocks. Participants began the IAT with 20 practice trials sorting the target images and 20 practice trials sorting positive and negative words. Next, participants completed two blocks of 20 and 40 critical trials, respectively, in which positive stimuli and smoking-related stimuli shared one response key and negative stimuli and non-smoking related stimuli shared another response key (or vice versa). Participants then practiced sorting target stimuli on 40 trials with a reversed response key assignment. Finally, participants completed a second set of blocks of 20 and 40 critical trials in which smoking-related stimuli shared a response key with negative and non-smoking related stimuli shared a response key with positive (or vice versa). If participants made an error in the categorization task, a red “X” appeared on the screen until participants provided the correct response. Latencies were recorded until a correct response was made. IAT-scores were calculated using the D2-algorithm [37], such that higher scores indicate a more positive implicit evaluation of smoking. Split-half reliability of the IAT score, calculated on the basis of an odd-even split, was high for each of the three groups of participants, *r*s = [.91 - .94].

Participants reported their explicit liking of smoking by responding to two separate questions. First, participants answered the question “Which of the following statements best describes you?” by using a 7-point response scale ranging from -3 (I strongly prefer smoking to not smoking) to +3 (I strongly prefer not smoking to smoking). Second, participants were asked to indicate to what extent they think smoking is positive or negative by giving their rating on a 7-point scale ranging from -3 (very negative) to +3 (very positive). We averaged these two ratings into one explicit rating score (Cronbach’s Alpha = .63).

After the implicit and explicit evaluation tasks, participants were asked to think back to the warning label that they saw at the beginning of the study and rate how effective they thought this label would be at reducing smoking. Participants gave their effectiveness ratings on a 6-point Likert scale ranging from 1 (not very effective at all) to 6 (extremely effective). Finally, participants were asked to indicate which of the following ten possible answers corresponded to their smoking behavior: ‘I never smoke’, ‘I smoke a couple of cigarettes per year’, ‘I smoke a couple of cigarettes per month’, ‘I smoke a couple of cigarettes per week’, ‘I smoke between 1 and 5 cigarettes per day’, ‘I smoke between 6 and 10 cigarettes per day’, ‘I smoke between 11 and 20 cigarettes per day’, ‘I smoke about one pack per day’, ‘I smoke between one and two packs per day’, ‘I smoke more than 2 packs per day’. Participants were categorized as non-smokers if they indicated the first option, as occasional smokers if they indicated smoking a couple of cigarettes per year/month/week and as daily smokers if they indicated any of the other options.

### Data analyses

To investigate effects of anti-smoking warning messages on IAT scores, explicit rating scores and effectiveness rating scores, we used linear mixed effects models (multilevel model analysis) as implemented in R package lme-4 [38]. Linear mixed effects models allow us to control for random effects of participants and items while assessing relevant (fixed) factors of interest (i.e., the effect of Warning Type: GW, text-only) and are very good at dealing with unbalanced data [39]. In line with standard recommendations, these linear mixed effects regression (lmer) analyses consisted of the following steps. We first defined a model with Warning Type as fixed factor and the grouping variable Participant as random factor (by-participant random intercept). Next, we tested if the inclusion of a random intercept for Smoking Behavior (i.e., participants’ answer to the smoking behavior question) or for Message Content (i.e., which of the text messages participants had read) significantly improved model fit. This allows us to control for variance in the scores that is due to differences in smoking behavior or due to the particular text-message participants had seen. There were 11 different text messages: nine text messages proposed by FDA (and included in GWs) and four currently used text messages, two of which are also part of the FDA messages. We also tested if the inclusion of an intercept for the factors Gender (male, female), Country of Residence (US, non-US), Race (White, Black, Other), Task Order (IAT first, explicit ratings first) or IAT Block Order (first IAT block involved categorizing positive and smoking or negative and smoking with the same key) improved model fit. We then tested if the inclusion of Age as a covariate improved model fit. In the next step we tested if random slopes for any of the fixed factors were additionally needed and whether the inclusion of Reading Time (i.e., the time it took participants to click ‘Continue’ to move on with the study when exposed to the screen displaying the anti-smoking warning) as a covariate improved model fit. On average, reading time was longer for participants who saw a GW (*M* = 21 s, *SD* = 24 s) than for participants who saw a text-only warning (*M* = 16 s, *SD* = 18 s), *t*(11368) = 7.43, *p* < .001, *d* = 0.22. Finally, analyses were performed for the best fitting model. For these analyses, we report all effects with *p* values < .10. Effects with a *p* value between .05 and .10 are also reported because these effects can be of interest to readers even though they are not to be considered significant according to standard levels of significance.

We supplemented lmer analyses with Bayesian analyses that were performed in accordance with standard procedures [40] with Cauchy prior width = .707. These analyses provide Bayes Factors (BFs) which give an indication of how strongly the data support either the null hypothesis (BF_0_; reflecting the absence of a significant effect) or the alternative hypothesis (BF_1_; reflecting the presence of a significant effect). Thus, they allow us to determine whether a non-significant finding can be considered support for the null hypothesis. We performed two separate one-sided Bayesian *t*-test analyses. More specifically, we tested (a) whether GWs produce better outcomes (i.e., *more negative* implicit and explicit evaluations of smoking) than text-only warnings (alternative hypothesis) or do not produce better outcomes (null hypothesis) and (b) whether GWs produce worse outcomes (i.e., *more positive* implicit and explicit evaluations of smoking) than text-only warnings (alternative hypothesis) or do not produce worse outcomes (null hypothesis). For the effectiveness ratings, Bayesian *t*-test analyses only tested whether GWs produce better outcomes (i.e., higher effectiveness ratings) than text-only warnings (alternative hypothesis) or do not produce better outcomes (null hypothesis). BFs between 1 and 3, between 3 and 10, and larger than 10, respectively designate ‘anecdotal evidence’, ‘substantial evidence’, and ‘strong evidence’ for either the null (BF0) or the alternative hypothesis (BF1) [41].

## Results

IAT scores and explicit rating scores indicated more negative evaluations of smoking for non-smokers (IAT: *M* = -0.77, *SD* = 0.40; explicit rating: *M* = -2.62, *SD* = 0.70) than for occasional smokers (IAT: *M* = -0.62, *SD* = 0.46; explicit rating: *M* = -1.44, *SD* = 1.13) and daily smokers (IAT: *M* = -0.56, *SD* = 0.53; explicit rating: *M* = -0.20, *SD* = 1.17), *t*s > 9.09, *p*s < .001. IAT scores showed a weak positive correlation with explicit rating scores, *r*(5831) = .25, *p* < .001, and with self-reported smoking behavior, *r*(5831) = .18, *p* < .001. The explicit rating scores showed a strong positive correlation with smoking behavior, *r*(5831) = .74, *p* < .001. The correlation between IAT scores and explicit rating scores was significantly higher for daily smokers (*r*[545] = .29) and occasional smokers (*r*[1165] = .29) than for non-smokers (*r*[4117] = .08), *z*s > 4.96, *p*s < .001.

### Lmer analyses on IAT and explicit rating scores

Lmer analyses were performed on standardized IAT and explicit rating scores for daily smokers, occasional smokers, and non-smokers. Scores were standardized by subtracting the mean implicit or explicit evaluation score from individual scores and dividing by the standard deviation of the score. Lmer models included the fixed factor of Evaluation Type (IAT score, explicit rating score) in addition to the factor of Warning Type. This allowed us to investigate whether Warning Type had a significantly stronger or weaker effect on implicit evaluations than on explicit evaluations.

#### Daily smokers

The best fitting model for daily smokers included only intercepts of Participant and IAT Block Order as random factors. Reading Time and Age were included as covariates. We observed a main effect of Evaluation Type, *χ*^2^(1) = 515.45, *p* < .001. In line with previous studies [42], results showed that smokers exhibit more positive explicit (*M* = 1.63, *SD* = 1.01) than implicit evaluations of smoking (*M* = 0.33, *SD* = 1.23). We also observed a main effect of Age, *χ*^2^(1) = 35.78, *p* < .001. Participants’ evaluation scores decreased by 0.18 for every ten years of age increase in participants. The main effect of Reading Time was marginally significant, *χ*^2^(1) = 2.85, *p* = .091. Participants’ evaluation scores decreased 0.04 for every ten seconds longer reading time. More importantly, we also observed a main effect of Warning Type, *χ*^2^(1) = 8.15, *p* = .004. Strikingly, evaluations of smoking were *more positive* when participants had seen a GW (*M* = 1.10, *SD* = 1.30) than when they had seen a text-only warning (*M* = 0.86, *SD* = 1.31). We did not observe a significant interaction with Evaluation Type, *χ*^2^(1) = 0.35, *p* = .55. Planned comparisons revealed a marginally significant effect of Warning Type on IAT scores, *t*(545) = 1.95, *p* = .052, *d* = 0.18, and a significant effect on explicit rating scores, *t*(545) = 2.65, *p* = .008, *d* = 0.25. Bayes factors provided strong evidence for the null hypothesis that, compared to text-only warnings, GWs do not produce *more negative* implicit, BF_0_ = 27.39, or explicit evaluations of smoking, BF_0_ = 34.74. They provided anecdotal evidence that GWs produce *more positive* implicit evaluations of smoking, BF_1_ = 1.27 and substantial evidence that GWs produce more positive explicit evaluations of smoking, BF_1_ = 6.21. We did not include Gender or Country of Residence in the final analyses because including these factors did not significantly improve model fit, *p*s > .40. We performed exploratory analyses which showed that the negative effect of GWs was (marginally) significant for male participants and female participants separately, and US and non-US participants separately, *p*s < .080”.

#### Occasional smokers

In the lmer analysis on evaluation scores of occasional smokers, random intercepts for Smoking Behavior and Participant were necessary as well as a random slope for Evaluation Type by Smoking Behavior. Reading Time and Age were included as covariates and Gender was included as a fixed factor. We observed a main effect of Evaluation Type, *χ*^2^(1) = 5.65, *p* = .017. Occasional smokers exhibited more positive explicit (*M* = 0.84, *SD* = 0.97) than implicit evaluations of smoking (*M* = 0.30, *SD* = 1.06). We also observed a main effect of Reading Time, *χ*^2^(1) = 11.98, *p* < .001. Participants’ evaluation scores decreased 0.04 for every ten seconds longer reading time. We also observed a main effect of Age, *χ*^2^(1) = 98.57, *p* < .001. Participants’ evaluation scores decreased by 0.20 for every ten years of age increase in participants. A main effect of Gender was also observed, *χ*^2^(1) = 11.98, *p* < .001, indicating that male participants exhibited more positive evaluations of smoking. The main effect of Warning Type was marginally significant, *χ*^2^(1) = 3.74, *p* = .053. Similar to results for daily smokers, evaluations of smoking were *more positive* when participants had seen a GW (*M* = 0.61, *SD* = 1.05) than when they had seen a text-only warning (*M* = 0.52, *SD* = 1.00). However, this main effect was qualified by three interactions. First, the interaction of Warning Type and Gender was marginally significant, *χ*^2^(1) = 3.77, *p* = .052. This interaction indicated that the main effect of Warning Type was significant for male participants, *χ*^2^(1) = 6.64, *p* = .009, but not for female participants, *χ*^2^(1) = 0.21, *p* = .64. Second, the interaction of Warning Type and Age was significant, *χ*^2^(1) = 4.19, *p* = .041. This interaction indicated that the main effect of Warning Type was bigger for older participants than for younger participants. Third, the interaction of Warning Type and Evaluation Type was marginally significant, *χ*^2^(1) = 3.12, *p* = .077. Planned comparisons revealed that standardized IAT scores were *more positive* when participants had seen a GW (*M* = 0.37, *SD* = 1.08) than when they had seen a text-only warning (*M* = 0.22, *SD* = 1.02), *t*(1165) = 2.52, *p* = .012, *d* = 0.17. We did not observe significant differences between explicit rating scores for participants who had seen a GW or a text-only warning, *t*(1165) = 0.35, *p* = .72, *d* = 0.02. Again, Bayes Factors indicated strong evidence for the null hypothesis that, compared to text-only warnings, GWs do not produce more negative implicit, BF_0_ = 47.40, or explicit evaluations of smoking, BF_0_ = 14.42. The Bayes Factors provided substantial evidence that GWs produce *more positive* implicit evaluations of smoking, BF_1_ = 3.38 and substantial evidence that GWs do not produce more positive explicit evaluations of smoking, BF_0_ = 9.91. We did not include Country of Residence in the final analyses because including this factor did not significantly improve model fit, *p* = .27. Exploratory analyses revealed that the negative effect of GWs was marginally significant for non-US participants and non-significant for US participants. This difference in effects was not significant, *p* = .58.

#### Non-smokers

The lmer model for non-smokers included the intercept of Participant as random factor. Reading Time and Age were included as covariates and Gender as fixed factor. In this model, we observed a main effect of Evaluation Type, *χ*^2^(1) = 298.70, *p* < .001. In contrast to the observed pattern for smokers, non-smokers exhibited more negative explicit (*M* = -0.40, *SD* = 0.60) than implicit evaluations of smoking (*M* = -0.11, *SD* = 0.92). We also observed a main effect of Reading Time, *χ*^2^(1) = 4.85, *p* = .004. Participants’ evaluation scores decreased 0.01 for every ten seconds longer reading time. We also observed a main effect of Gender, *χ*^2^(1) = 42.56, *p* < .001, indicating that male participants exhibited more positive evaluations of smoking. We also observed a main effect of Age, *χ*^2^(1) = 239.18, *p* < .001. Participants’ evaluation scores decreased by 0.10 for every ten years of age increase in participants. We did not observe a main effect of Warning Type, *χ*^2^(1) = 0.13, *p* = .72, nor an interaction with Evaluation Type, *χ*^2^(1) = 1.89, *p* = .17. Planned comparisons revealed no significant differences in IAT scores or explicit rating scores for participants who had seen a GW (*M* = -0.25, *SD* = 0.79) compared to participants who had seen a text-only warning (*M* = -0.26, *SD* = 0.80), *t*s < 1.44, *p*s > 15, *ds* < 0.06. Bayes Factors indicated substantial evidence for the null hypothesis that GWs do not produce *more negative* implicit evaluations compared to text-only warnings, BF_0_ = 4.91, and strong evidence for the null hypothesis that GWs do not produce more negative explicit evaluations compared to text-only warnings, BF_0_ = 39.66. The Bayes Factors also provided strong evidence that GWs do not produce *more positive* implicit or explicit evaluations of smoking, BF_0s_ > 13.81. We did not include Country of Residence in the final analyses because including this factor did not significantly improve model fit, *p* = .10. Exploratory analyses revealed no effect of Warning Type for US or non-US participants, *p*s > .34

### Lmer analyses on effectiveness rating scores

Effectiveness rating scores showed a weak negative correlation with self-reported smoking behavior, *r*(5831) = -.14, *p* < .001, indicating that participants who reported that they smoke more, rated the anti-smoking warnings as less effective. This correlation was also observed when we excluded the data of the participants who indicated that they did not smoke, *r*(1712) = -.14, *p* < .001. We also observed a weak negative correlation of effectiveness rating scores with IAT scores, *r*(5831) = -.06, *p* < .001, and with explicit rating scores, *r*(5831) = -.16, *p* < .001.

#### Daily smokers

We fitted an lmer model for effectiveness ratings of daily smokers. The best fitting model included random intercepts for the factors Smoking Behavior and Message Content and a fixed effect of Gender. We observed a main effect of Gender, *χ*^2^(1) = 5.38, *p* = .020, indicating that male participants gave lower effectiveness ratings in general. The effect of Warning Type was marginally significant, *χ*^2^(1) = 3.04, *p* = .081, *d* = 0.15. Participants gave descriptively higher effectiveness ratings for GWs (*M* = 1.97, *SD* = 1.30) than for text-only warnings (*M* = 1.84, *SD* = 1.31). The obtained Bayes Factor indicated that results provide anecdotal evidence for the null hypothesis (i.e., that daily smokers *do not* rate GWs as more effective than text-only warnings, BF_0_ = 1.38).

#### Occasional smokers

The lmer model included random intercepts for the factors Smoking Behavior and Message Content and a fixed effect of Gender. We observed a main effect of Gender, *χ*^2^(1) = 21.88, *p* < .001, indicating that male participants gave lower effectiveness ratings in general. In contrast to results for daily smokers, we also observed a significant main effect of Warning Type, *χ*^2^(1) = 26.97, *p* < .001, *d* = 0.33. Effectiveness ratings were higher for GWs (*M* = 2.38, *SD* = 1.05) than for text-only warnings (*M* = 2.06, *SD* = 1.00). The obtained Bayes Factor indicated that results provide strong evidence for the alternative hypothesis (i.e., that occasional smokers rate GWs as more effective than text-only warnings, BF_1_ = 21872).

#### Non-smokers

The lmer model for non-smokers included a random intercept for Message Content, a random slope for Warning Type by Message Content and fixed effects of Gender and Age. We observed a main effect of Gender, *χ*^2^(1) = 20.64, *p* < .001, indicating that male participants gave lower effectiveness ratings in general, and a main effect of Age, *χ*^2^(1) = 5.52, *p* = .019, indicating that effectiveness scores decreased by 0.03 for every ten years of age increase in participants. The main effect of Warning Type was significant, *χ*^2^(1) = 38.68, *p* < .001, *d* = 0.58, indicating that non-smokers rated GWs (*M* = 2.65, *SD* = 0.79) as more effective than text-only warnings (*M* = 2.22, *SD* = 0.80), BF_1_ > 10^5^.

## Discussion

In the current study, we examined whether GWs elicit more negative implicit evaluations of smoking than text-only warnings. To this end, we measured smokers’ and non-smokers’ implicit evaluations of smoking with an IAT after they had viewed a GW or a text-only warning. Results provided strong evidence that, even though (occasional) smokers and non-smokers rate GWs as more effective than text-only warnings, they do not exhibit more negative implicit evaluations of smoking after viewing a GW than after viewing a text-only warning. If anything, GWs seemed to produce worse effects than text-only warnings in daily and occasional smokers (i.e., *more positive* implicit evaluations of smoking). Similarly, GWs also produced only unwanted effects on explicit evaluations of smoking (i.e., *more positive* explicit evaluations of smoking compared to text-only warnings in daily smokers).

### GWs do not elicit more negative implicit evaluations of smoking

Given the strong evidence for the effectiveness of GWs in changing smoking behavior (e.g., [9]), it is surprising that we did not observe a negative shift in implicit evaluations of smoking for participants who viewed GWs. A number of possible explanations might account for this result. First, psychological reactance might have played a role in our studies. It is often argued that GWs can initiate a freedom threat in smokers and can therefore lead smokers to experience fear or cognitive dissonance [43]. As a result, smokers may become motivated to reduce these negative feelings evoked by GWs. This may be achieved more easily by raising counterarguments against the unelaborated warning messages than by changing their smoking behavior. This might impact spontaneous responses to smoking-related stimuli (e.g., because information in-line with the idea that participants like smoking is now more active in memory), making IAT scores of smoking more positive. In accordance with this explanation, previous studies have provided strong evidence that (a) GWs elicit greater reactance in smokers than text-only warnings (mean *d* = 0.50, see [3]) and (b) the inclusion of graphic images in cigarette warnings leads to more negative thoughts about cigarette warnings [10]. Moreover, evidence suggest that such reactance processes can sometimes lead to unwanted changes in implicit evaluations of smoking [29]. Further in-line with this idea, we recently performed a study in which 1302 online participants read a fear-inducing text-only warning (an FDA warning) or a text-only warning that focused on the positive effects of quitting smoking (e.g., quitting smoking restores lung functioning). We found that participants who saw the fear-inducing warnings came up with more counter-arguments and this produced more positive implicit evaluations of smoking. In that study, we also found that male participants exhibited higher psychological reactance (as often observed: e.g.:[44]) and generated more counter-arguments. This accords with our observation in the current study that implicit evaluations of male participants were more negatively impacted by GWs than female participants (for occasional smokers).

A second explanation is that implicit evaluations simply cannot change on the basis of a one-time exposure to cigarette pack warnings. Importantly, however, the current study does provide evidence for the malleability of implicit evaluations of smoking as the result of a single viewing of GWs. However, results indicate that when GWs have an influence, it is in the unintended direction. Specifically, compared to text-only warnings, GWs produced *more positive* implicit evaluations of smoking for daily and occasional smokers. This accords with recent studies showing that implicit evaluations in general, and implicit evaluations of smoking in particular, can be readily changed via the one-time viewing of verbal information and supports evaluation models that incorporate this possibility in their models [32,33]. Note that the observed negative reaction to GWs might be only an initial (spontaneous) reaction that is very momentary. It is possible that changes in implicit evaluations (and related changes in behavior) might be more positive after repeated (or longer) exposures to GWs. In line with this idea, a recent study found a (small) beneficial effect of GWs compared to text-only messages on implicit evaluations of smoking as measured with the IAT in a sample of young adult smokers [45]. Importantly, in that study, participants saw the nine FDA-proposed GWs four times each in a six minute time span. It is possible that this bombarding of participants with GWs is more effective for changing implicit evaluations in the intended direction (e.g., because participants do not have the time to argue against each warning).

A third explanation is that other (within-participant) factors influenced implicit evaluations and that these factors were not matched between the text conditions. We probed several possible moderators of implicit evaluations and found that some of these factors influenced observed effects (e.g., age and gender) while others did not (e.g., country of residence and race). However, it is possible that other factors, that were not measured, also biased implicit evaluations and might therefore have influenced the observed results (e.g., trait reactance, or time since a person’s last cigarette: [46]). It is also possible that procedural factors influenced implicit evaluations and precluded positive effects of GWs. For instance, in the current study we told participants to look at warnings that the FDA was considering to put on cigarette packs. It is possible that this amplified psychological reactance in some participants, which, in turn, influenced implicit evaluations of smoking. Note, however, that instructions were similar for participants who viewed GWs or text-only warnings, so overall reactance due to instructions cannot explain differences between the two text conditions. Relatedly, it is possible that effects were biased by the fact that GWs were presented in a different manner than is typically the case (i.e., on a webpage rather than on a cigarette pack). Furthermore, participants were given the instructions to review the message carefully which might have further biased results compared to actual exposure in real life. For instance, negative aspects might be more easily reported when consciously reviewing these messages in an on-line study (e.g., to accord with inferred experimenter expectations). Future studies might examine effects of warnings in more ecologically valid situations such as when messages are presented on cigarette packs or when participants are more cognitively busy.

A final explanation is that IAT scores were changed (in the unintended direction) due to GWs, but that this change does not reflect a change in actual liking of smoking (and hence is unlikely to influence smoking behavior). Indeed, there is evidence to suggest that IAT performance can be influenced by non-attitudinal factors such as participants’ recoding of IAT categories [47] or extra-personal knowledge (e.g., knowledge that a person has about societal views but regards as irrelevant for his or her own feelings about the attitude object: [48]). This has also been proposed as an explanation for why some studies do not find any relation between (changes in) IAT scores and important smoking-related outcomes (e.g.: [49]). It is therefore essential that future work examines effects of cigarette pack warnings on other implicit evaluation measures as well and examines how changes in these measures relate to changes in other smoking-related behavior.

### GWs do not elicit more negative explicit evaluations of smoking

For the sake of comparison, the current study also examined effects of GWs on more controlled, explicit evaluations of smoking. Results provide strong evidence that, similar to implicit evaluations, explicit evaluations also do not become more negative when smokers or non-smokers view a GW compared to a text-only warning. This contrasts with the conclusion of [3] who stated that “relative to text-only warnings, pictorial warnings elicit more negative smoking attitudes” (p. 1). This discrepancy may again be related to the fact that participants in our study only needed to watch the GWs one time and for a short period of time. Our failure to find beneficial effects of GWs on explicit ratings of smoking in *smokers* accords with the idea that smokers’ explicit evaluations of smoking are held with great certainty and are therefore very resistant to persuasive attempts [30]. Likewise, *non-smokers* may have formed a clear image of the negativity of smoking and this does not change when they are confronted with a graphic image (added to a cigarette warning). Given the strongly negative rating of smoking for non-smokers, this might also be a floor effect.

Similar to results for implicit evaluations, however, we also found some evidence for unwanted effects of GWs on explicit evaluations of smoking. Results indicated that GWs produced more positive explicit evaluations of smoking in daily smokers. In contrast to implicit evaluations, however, occasional smokers’ explicit evaluations of smoking did not become more positive after viewing GWs. This discrepancy accords with the observation that occasional smokers but not daily smokers rated GWs as more effective than text-only warnings. These results suggest that reactance may more easily present itself on controlled measures such as effectiveness and liking ratings for daily smokers than for occasional smokers. Occasional smokers might feel more uncertain about their reasons for smoking and therefore show less reactance on these explicit measures. After viewing the anti-smoking warnings, occasional smokers might more strongly agree that smoking is bad and that therefore fear-evoking messages are needed (even though viewing these GWs does not reduce their spontaneous liking of smoking). This might suggest that implicit measures of evaluation may sometimes capture important (reactance-based) changes that are more difficult to capture with explicit evaluation measures (see also [29]).

### Implications

The current findings might help explain why GWs do not always produce beneficial effects on smoking behavior (even when they change intentions to quit smoking) [11]. For instance, addictive behaviors such as smoking may have a strong automatic component and changes in implicit evaluation may therefore be important for reducing smoking behavior [15–16] (e.g., by establishing a reduction in craving for cigarettes [50] or by improving self-efficacy [51]). GWs may typically produce better effects than text-only warnings on controlled outcomes such as intentions to quit [3, 52], which prompts smoking cessation in general (e.g., [8]). However, some smokers know the negative consequences of smoking very well, and they may even consider them a good reason for quitting, yet they still persist in their smoking habit. In those cases, it might be important to produce changes in automatic evaluative reactions to smoking. When GWs lead to psychological reactance, however, these reactions might become more positive. Hence, our results suggest that, in order to find more beneficial effects of GWs it might be important to use GWs that are less (construed as) fear-evoking and therefore lead to less reactance. In accordance with this idea, one study found that smokers indicated a greater amount of quitting activity in the weeks following exposure to GWs when these GWs were less reactance-evoking because they included photographs and personal details of real people whose health has been affected by smoking [53]. Our results further suggest that (reactant) responses to GWs might depend on specific characteristics of the target group. For instance, when it comes to occasional smokers, it seems that older, male, smokers might react more negatively to GWs.

For non-smokers, it may also be of importance to make implicit evaluations of smoking more negative. More negative automatic reactions towards smoking may encourage them not to pick up smoking. For instance, a strong implicit disliking of smoking might overcome social influence when non-smokers have to decide whether or not to light up a cigarette. However, it can also lead them to express these spontaneous reactions which could influence their reactions to smokers. As our results indicate, however, confronting non-smokers with GWs does not produce more negative implicit evaluations in non-smokers than presenting text-only warnings.

It is important to note that our results do not show that GWs have no benefit in general. Rather, they may provide information that suggests that it may be of importance to integrate knowledge about psychological reactance when designing new anti-smoking warnings. Moreover, because of the immediate impact that warnings may have on implicit evaluations, it might be beneficial to examine effects on implicit evaluations to improve the effectiveness of these warnings. A possible step when designing new anti-smoking warnings may be to establish which specific anti-smoking messages (in combination with which specific graphic images) produce the strongest desired effects on implicit evaluations. To provide a first indication, we performed analyses investigating the separate effects of the 11 different text messages that were used in this study (presented with and without graphics). These analyses are reported in the online supplement and indicated, for instance, that the message ‘cigarettes cause cancer’ produces more negative evaluations of smoking than the message ‘tobacco smoke causes fatal lung disease in nonsmokers’ for daily smokers. One possible reason for this might be that the latter message can be more easily discarded (e.g., when a smoker argues that they don’t smoke near nonsmokers). Note that for non-smokers the latter message was actually the second *best* message (which might be because this message is more self-relevant) only preceded by ‘smoking can kill you’.

### Concluding remarks

In sum, the current findings suggest that the one-time viewing of GWs does not have any beneficial effect on implicit (or explicit) evaluations of smoking in smokers or non-smokers. Although GWs were *perceived* as being more effective by non-smokers and occasional smokers, these ratings of effectiveness were inaccurate with regard to immediate effects on evaluations of smoking. Where significant differences in implicit (and explicit) evaluations of smoking occurred, they were in the direction of evaluations being *more positive* after participants viewed GWs. Future studies are required to further test effects of GWs on implicit evaluations of smoking as measured with other measures of implicit evaluation and to test their relation with actual smoking behavior.

## References

[1] World Health Organization. WHO report on the global tobacco epidemic; 2017. Available from: http://www.who.int/tobacco/global_report/2017/en/

[2] Witte K, Allen M. A meta-analysis of fear appeals: Implications for effective public health campaigns. Health Educ Behav. 2000; 27: 591–616. doi: 10.1177/109019810002700506 11009129 10.1177/109019810002700506

[3] Noar S, Hall M, Francis D, Ribisl K, Pepper J, Brewer N. Pictorial cigarette pack warnings: A meta-analysis of experimental studies. Tob Control. 2016; 25: 341–354. doi: 10.1136/tobaccocontrol-2014-051978 25948713 10.1136/tobaccocontrol-2014-051978 PMC4636492

[4] Hammond D. Health warning messages on tobacco products: a review. Tob. Control, 2011; 20; 327–337. doi: 10.1136/tc.2010.037630 21606180 10.1136/tc.2010.037630

[5] Fong GT, Hammond D, Jiang Y, Li Q, Quah AC, Driezen P, et al Perceptions of tobacco health warnings in China compared with picture and text-only health warnings from other countries: an experimental study. Tob Control 2010;19:I69–77. doi: 10.1136/tc.2010.036483 20935200 10.1136/tc.2010.036483 PMC2976466

[6] Hammond D, Fong GT, Borland R, Cummings KM , McNeill A, Driezen P. Text and graphic warnings on cigarette packages—Findings from the International Tobacco Control Four Country Study. Am J Prev Med 2007; 32: 202–209 doi: 10.1016/j.amepre.2006.11.011 17296472 10.1016/j.amepre.2006.11.011 PMC1868456

[7] White V, Webster B, Wakefield M. Do graphic health warning labels have an impact on adolescents' smoking-related beliefs and behaviours? Add 2008; 103: 1562–1571 10.1111/j.1360-0443.2008.02294.x 18783508

[8] Brewer NT, Hall MG, Noar SM, Parada H, Stein-Seroussi A, Bach LE, et al Effect of pictorial cigarette pack warnings on changes in smoking behavior a randomized clinical trial. JAMA Intern Med. 2016; 176: 905–912. doi: 10.1001/jamainternmed.2016.2621 27273839 10.1001/jamainternmed.2016.2621 PMC5458743

[9] Noar SM, Francis DB, Bridges C, Sontag JM, Ribisl KM, Brewer NT. The impact of strengthening cigarette pack warnings: systematic review of longitudinal observational studies. Soc Sci Med. 2016; 164: 118–129. doi: 10.1016/j.socscimed.2016.06.011 27423739 10.1016/j.socscimed.2016.06.011 PMC5026824

[10] LaVoie N, Quick B, Riles J, Lambert N. Are graphic cigarette warning labels an effective message strategy? A test of psychological reactance theory and source appraisal. Communic Res. 2015 doi: 10.1177/0093650215609669

[11] Monarrez-Espino J, Bojing L, Greiner F, Bremberg S, Galanti R. Systematic review of the effect of pictorial warnings on cigarette packages in smoking behaviour. Am J Public Health. 2014; 104: 11–30. doi: 10.2105/AJPH.2013.301471 25122019 10.2105/AJPH.2014.302129 PMC4167086

[12] Ruiter R, Kok G. Saying is not (always) doing: Cigarette warnings labels are useless. Eur J Public Health. 2005; 15: 329–330. 10.1093/eurpub/cki095 15985460

[13] Webb T, Sheeran P. Does changing behavioral intentions engender behavior change? A meta-analysis of the experimental evidence. Psychol Bull. 2006; 132: 249–268. doi: 10.1037/0033-2909.132.2.249 16536643 10.1037/0033-2909.132.2.249

[14] Wood W, Quinn J, Kashy D. Habits in everyday life: The thought and feel of action. J Pers Soc Psychol. 2002; 83: 1281–1297. 12500811

[15] Sherman S, Chassin L, Presson C, Seo D., Macy J. The intergenerational transmission of implicit and explicit attitudes toward smoking: Predicting adolescent smoking initiation. J Exp Soc Psychol. 2009; 45: 313–319. doi: 10.1016/j.jesp.2008.09.012 20126293 10.1016/j.jesp.2008.09.012 PMC2703492

[16] Chassin L, Presson C, Sherman S, Seo D, Macy J. Implicit and explicit attitudes predict smoking cessation: Moderating effects of experienced failure to control smoking and plans to quit. Psychol Addict Behav. 2010; 24: 670–679. doi: 10.1037/a0021722 21198227 10.1037/a0021722 PMC3059811

[17] Kahler C, Daughters S, Leventhal A, Gwaltney C, Palfai T. Implicit Associations between Smoking and Social Consequences Among Smokers in Cessation Treatment. Behav Res Ther. 2007; 45: 2066–2077. doi: 10.1016/j.brat.2007.03.004 17448442 10.1016/j.brat.2007.03.004 PMC1986791

[18] Rooke S, Hine D, Thorsteinsson E. Implicit cognition and substance use: A meta-analysis. Addict Behav. 2008; 33, 1314–1328. doi: 10.1016/j.addbeh.2008.06.009 18640788 10.1016/j.addbeh.2008.06.009

[19] Lee H, Addicott M, Martin L, et al Implicit Attitudes and Smoking Behavior in a Smoking Cessation Induction Trial. Nicotine Tob Res. 2016 doi: 10.1093/ntr/ntw259 10.1093/ntr/ntw259 PMC5896513 27679606

[20] Macy J, Chassin L, Presson C. The Association Between Implicit and Explicit Attitudes Toward Smoking and Support for Tobacco Control Measures. Nicotine Tob Res. 2013; 15: 291–296. doi: 10.1093/ntr/nts117 22581941 10.1093/ntr/nts117 PMC3524067

[21] Hughes S, Barnes-Holmes D, De Houwer J. The Dominance of Associative Theorizing in Implicit Attitude Research: Propositional and Behavioral Alternatives. Psychol Rec. 2011; 61: 465–496.

[22] Fazio R, Sanbonmatsu D, Powell M, Kardes F. On the automatic activation of attitudes. J Pers Soc Psychol. 1986; 50: 229–238. 3701576 10.1037//0022-3514.50.2.229

[23] Smith E, DeCoster J. Dual-process models in social and cognitive psychology: Conceptual integration and links to underlying memory systems. Pers Soc Psychol Rev, 2000; 4: 108–131.

[24] Măgurean S, Constantin T, Savaa F. The indirect effect of evaluative conditioning on smoking. J Subst Use. 2016; 21: 198–203.

[25] Macy J, Chassin L, Presson C, Sherman J. Changing implicit attitudes toward smoking: results from a web-based approach-avoidance practice intervention. J Behav Med. 2015; 38: 143–52. doi: 10.1007/s10865-014-9585-2 25059750 10.1007/s10865-014-9585-2 PMC4302002

[26] Horcajo J, Briñol P, Petty R. Consumer persuasion: Indirect change and implicit balance. Psychol Market. 2010; 27: 938–963.

[27] Mann T, Cone J, Ferguson M. Social-psychological evidence for the effective updating of implicit attitudes. Behav Brain Sci. 2015; 38: 32–33. 10.1017/S0140525X14000223 26050678

[28] Smith C, De Houwer J, Nosek B. Consider the source: Persuasion of implicit evaluations is moderated by source credibility. Pers Soc Psychol Bull. 2013; 39: 193–205. doi: 10.1177/0146167212472374 23386656 10.1177/0146167212472374

[29] Smith C, De Houwer J. Hooked on a feeling: Affective anti-smoking messages are more effective than cognitive messages at changing implicit evaluations of smoking. Front Psychol, 2015; 6:1488 doi: 10.3389/fpsyg.2015.01488 26557099 10.3389/fpsyg.2015.01488 PMC4617384

[30] Rydell R, Sherman S, Boucher K, Macy J. The role of motivational and persuasive message factors in changing implicit attitudes toward smoking. Basic Appl Soc Psychol. 2012; 34: 1–7. 10.1080/01973533.2011.637847 PMC3630374 23606782

[31] De Houwer J. Using the Implicit Association Test does not rule out an impact of conscious propositional knowledge on evaluative conditioning. Learn Motiv. 2006; 37: 176–187.

[32] De Houwer J. A Propositional Model of Implicit Evaluation. Soc Personal Psychol Compass. 2014; 8: 342–353.

[33] Gawronski B, Bodenhausen G. Associative and propositional processes in evaluation: An integrative review of implicit and explicit attitude change. Psychol Bull. 2006; 132: 692–731. doi: 10.1037/0033-2909.132.5.692 16910748 10.1037/0033-2909.132.5.692

[34] Gawronski B, Bodenhausen G. The associative–propositional evaluation model: Operating principles and operating conditions of evaluation In Sherman J. W., Gawronski B., & Trope Y. (Eds.), Dual-process theories of the social mind, 2014: 188–203. New York, NY: Guilford Press.

[35] Greenwald A, McGhee D, Schwartz J. Measuring individual differences in implicit cognition: the implicit association test. J Pers Soc Psychol. 1998; 74: 1464–1480. 9654756 10.1037//0022-3514.74.6.1464

[36] Blanton H, Snyder L, Strauts E, Larson J. Effect of Graphic Cigarette Warnings on Smoking Intentions in Young Adults. PLoS ONE. 2014; 9(5): e96315 doi: 10.1371/journal.pone.0096315 24806481 10.1371/journal.pone.0096315 PMC4012950

[37] Greenwald A, Nosek B, Banaji M. Understanding and using the Implicit Association Test: I. An improved scoring algorithm. J Pers Soc Psychol. 2003; 85: 197–216. 12916565 10.1037/0022-3514.85.2.197

[38] Bates D, Maechler M, Bolker B, Walker S. lme4: Linear mixed-effects models using Eigen and S4. R package version 1.0–6. 2014. http://CRAN.R-project.org/package=lme4

[39] Baayen R, Davidson D, Bates D. Mixed-effects modeling with crossed random effects for subjects and items. J Mem Lang. 2008; 59: 390–412.

[40] Rouder J, Speckman P, Sun D, Morey R, Iverson G. Bayesian t tests for accepting and rejecting the null hypothesis. Psychon Bull & Rev. 2009; 16: 225–237. 19293088 10.3758/PBR.16.2.225

[41] Jeffreys H. Theory of Probability. 1961 Oxford: Oxford University Press.

[42] Sherman S, Rose J, Koch K, Presson C, Chassin L. Implicit and explicit attitudes toward cigarette smoking: The effects of context and motivation. Soc Clin Psychol. 2003; 22: 13–39.

[43] de Hoog N, Stroebe W, de Wit J. The impact of vulnerability to and severity of a health risk on processing and acceptance of fear-arousing communications: A meta-analysis. Rev Gen Psychol. 2007; 11: 258–285.

[44] Seeman E., Buboltz W, Jenkins S, Soper B & Woller K. Ethnic and gender differences in psychological reactance: The importance of reactance in multicultural counseling. Couns Psych Quart. 2004; 17, 167–176.

[45] Macy J, Chassin L, Presson C, Yeung E. Exposure to graphic warning labels on cigarette packages: Effects on implicit and explicit attitudes towards smoking among young adults. Psychol Health. 2016; 31: 349–63. doi: 10.1080/08870446.2015.1104309 26442992 10.1080/08870446.2015.1104309 PMC4767655

[46] Mogg K, Field M, Bradley B. Attentional and approach biases for smoking cues in smokers: An investigation of competing theoretical views of addiction. Psychopharmacology, 2005; 180: 333–341. doi: 10.1007/s00213-005-2158-x 15696322 10.1007/s00213-005-2158-x

[47] Rothermund K, Teige-Mocigemba S, Gast A, Wentura D. Minimizing the influence of recoding in the Implicit Association Test: The Recoding-Free Implicit Association Test (IAT-RF). Quart J Exp Psych. 2009; 62, 84–98. 10.1080/17470210701822975 18609400

[48] Olson M, Fazio R. Reducing the influence of extra-personal associations on the Implicit Association Test: Personalizing the IAT. J Pers Soc Psych. 2004; 86, 653–667. 10.1037/0022-3514.86.5.653 15161392

[49] Spruyt A, Lemaigre V, Salhi B, Van Gucht D, Tibboel H, Van Bockstaele B, et al Implicit attitudes towards smoking predict long-term relapse in abstinent smokers. Psychopharmacology. 2015; 232: 2551–2561. doi: 10.1007/s00213-015-3893-2 25761836 10.1007/s00213-015-3893-2

[50] Waters A, Carter B, Robinson J, Wetter D, Lam C, Cinciripini P. Implicit attitudes to smoking are associated with craving and dependence. Drug Alcohol Dep. 2007; 91: 178–186. 10.1016/j.drugalcdep.2007.05.024 PMC3275631 17658701

[51] Romer D, Peters E, Strasser A, Langleben D. Desire versus Efficacy in Smokers’ Paradoxical Reactions to Pictorial Health Warnings for Cigarettes. PLoS ONE. 2013; 8(1): e54937 doi: 10.1371/journal.pone.0054937 23383006 10.1371/journal.pone.0054937 PMC3558430

[52] Evans A, Peters E, Strasser A, Emery L, Sheerin K, Romer D. Graphic Warning Labels Elicit Affective and Thoughtful Responses from Smokers: Results of a Randomized Clinical Trial. PLoS ONE. 2015; 10(12): e0142879 doi: 10.1371/journal.pone.0142879 26672982 10.1371/journal.pone.0142879 PMC4684406

[53] Brennan E, Maloney EK, Ophir Y, Cappella JN. Potential Effectiveness of Pictorial Warning Labels That Feature the Images and Personal Details of Real People. Nicotine Tob Res. 2016; pii: ntw319 10.1093/ntr/ntw319 PMC5896434 27932628

